# 3-Ethyl-3-hy­droxy-8-meth­oxy­quinoline-2,4(1*H*,3*H*)-dione monohydrate

**DOI:** 10.1107/S1600536812043280

**Published:** 2012-10-24

**Authors:** Stanislav Kafka, Andrej Pevec, Karel Proisl, Roman Kimmel, Janez Košmrlj

**Affiliations:** a Department of Chemistry, Faculty of Technology, Tomas Bata University in Zlin, Zlin 76272, Czech Republic; b Faculty of Chemistry and Chemical Technology, University of Ljubljana, SI-1000 Ljubljana, Slovenia

## Abstract

In the title hydrate, C_12_H_13_NO_4_·H_2_O, the piperidine ring that is fused to the benzene ring is in a sofa conformation with the chiral C atom lying 0.4084 (18) Å out of the plane of the nine fused-ring atoms. In the crystal, O—H⋯O and N—H⋯O hydrogen bonds link the organic mol­ecules and water mol­ecules into chains running along the *b*-axis direction. The chains are further connected into layers parallel to the *bc* plane by π–π inter­actions between inversion-related benzene rings [centroid–centroid distance = 3.8846 (9) Å].

## Related literature

For methods of preparation of 3-alkyl- or 3-aryl-3-hy­droxy­quinoline-2,4-diones by oxidation of the corresponding 3-alkyl- or 3-aryl­quinolin-2-ones, see: Stadlbauer & Kappe (1982 ▶); Stadlbauer *et al.* (1992 ▶). For naturally occurring 3-hy­droxy­quinoline-2,4-diones, see: Neuenhaus & Budzikiewicz (1979 ▶); Luo *et al.* (2009 ▶). For the biological activity of 3-hy­droxy­quinoline-2,4-diones, see: Prisyazhnyuk *et al.* (1984 ▶); Luo *et al.* (2009 ▶). For a related structure, see: Kafka *et al.* (2012 ▶).
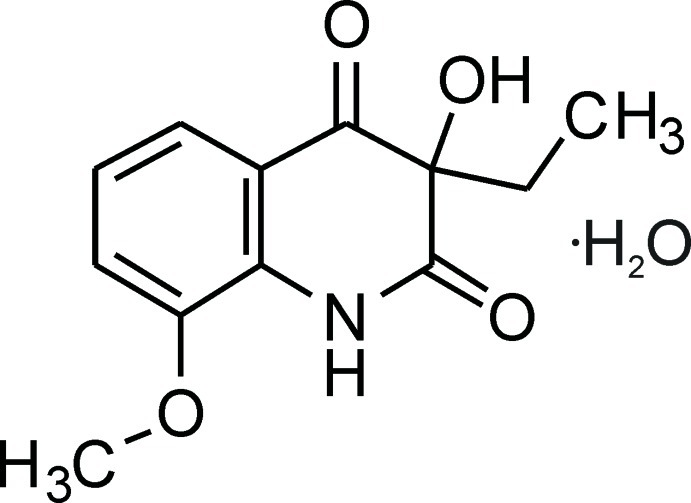


## Experimental

### 

#### Crystal data

C_12_H_13_NO_4_·H_2_O
*M* *_r_* = 253.25
Orthorhombic, 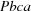

*a* = 16.5055 (4) Å
*b* = 8.8068 (2) Å
*c* = 16.6690 (4) Å
*V* = 2423.02 (10) Å^3^
*Z* = 8
Mo *K*α radiation
μ = 0.11 mm^−1^
*T* = 293 K
0.50 × 0.25 × 0.20 mm

#### Data collection

Nonius KappaCCD area-detector diffractometer
Absorption correction: multi-scan (*SCALEPACK*; Otwinowski & Minor, 1997 ▶) *T*_min_ = 0.948, *T*_max_ = 0.979
5200 measured reflections
2779 independent reflections
1963 reflections with *I* > 2σ(*I*)
*R*_int_ = 0.019

#### Refinement

*R*[*F* ^2^ > 2σ(*F* ^2^)] = 0.044
*wR*(*F* ^2^) = 0.127
*S* = 1.03
2779 reflections
175 parameters
3 restraints
H atoms treated by a mixture of independent and constrained refinement
Δρ_max_ = 0.26 e Å^−3^
Δρ_min_ = −0.23 e Å^−3^

### 

Data collection: *COLLECT* (Nonius, 1998 ▶); cell refinement: *DENZO* and *SCALEPACK* (Otwinowski & Minor, 1997 ▶); data reduction: *DENZO* and *SCALEPACK*; program(s) used to solve structure: *SIR92* (Altomare *et al.*, 1994 ▶); program(s) used to refine structure: *SHELXL97* (Sheldrick, 2008 ▶); molecular graphics: *PLATON* (Spek, 2009) ▶ and *DIAMOND* (Brandenburg, 1999 ▶); software used to prepare material for publication: *WinGX* (Farrugia, 1999 ▶).

## Supplementary Material

Crystal structure: contains datablock(s) I, global. DOI: 10.1107/S1600536812043280/tk5162sup1.cif

Structure factors: contains datablock(s) I. DOI: 10.1107/S1600536812043280/tk5162Isup2.hkl

Supplementary material file. DOI: 10.1107/S1600536812043280/tk5162Isup3.cml

Additional supplementary materials:  crystallographic information; 3D view; checkCIF report

## Figures and Tables

**Table 1:** Hydrogen-bond geometry (Å, °)

*D*—H⋯*A*	*D*—H	H⋯*A*	*D*⋯*A*	*D*—H⋯*A*
O3—H3⋯O1^i^	0.82	2.35	2.9430 (17)	130
N1—H1*N*⋯O1*W*	0.86 (1)	2.01 (2)	2.8438 (19)	163 (2)
O1*W*—H1*W*⋯O2^ii^	0.92 (2)	2.07 (2)	2.9425 (19)	158 (2)
O1*W*—H2*W*⋯O3^iii^	0.92 (2)	2.15 (2)	2.9401 (19)	144 (2)

Symmetry codes: (i) 
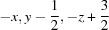
; (ii) 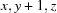; (iii) 
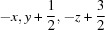
.

**Fig. 1.**
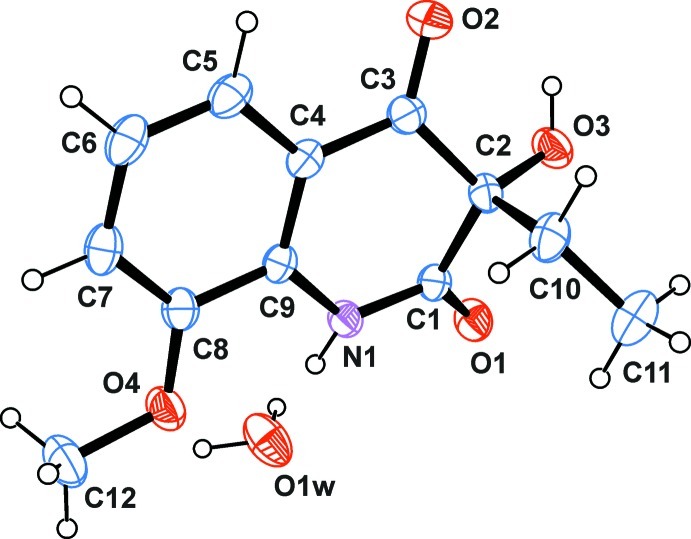
A view of the title compound showing the atom-numbering scheme. Displacement ellipsoids are drawn at the 30% probability level.

**Fig. 2.**
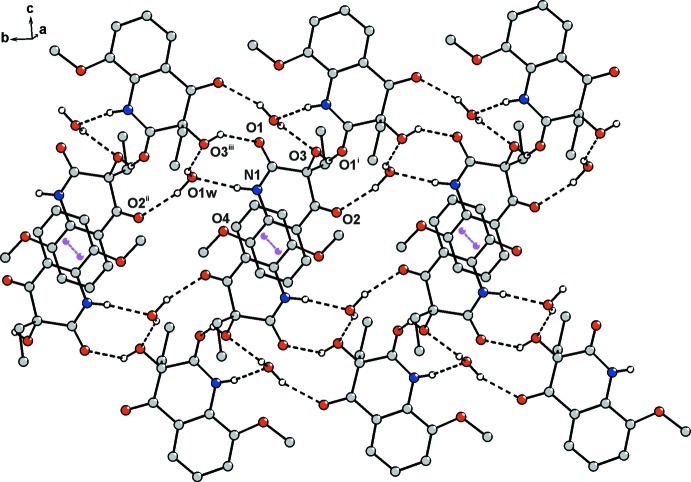
The packing of (I), with the O—H···O and N—H···O hydrogen bonds, and π—π interactions denoted by dashed lines. Hydrogen atoms not involved in hydrogen bonding have been omitted for clarity. [Symmetry codes: (i) -x, y - 1/2, -z + 3/2; (ii) x, y + 1, z; (iii) -x, y + 1/2, -z + 3/2]

## supplementary crystallographic information

### Comment

3-Alkyl- and 3-aryl-3-hydroxyquinoline-2,4-diones have been prepared by oxidation of the corresponding 3-alkyl- or 3-arylquinolin-2-ones by means of oxygen under UV irradiation (Stadlbauer & Kappe, 1982), *m*-chloroperoxybenzoic acid (Stadlbauer & Kappe, 1982), hydrogen peroxide (Stadlbauer & Kappe, 1982), nitric acid (Stadlbauer *et al.*, 1992), or peracetic acid (Stadlbauer *et al.*, 1992). Several 3-hydroxyquinoline-2,4-diones were isolated from bacteria *Pseudomonas aeruginosa* (Neuenhaus & Budzikiewicz, 1979) and from stem bark of *Micromelum falcatum* (Luo *et al.*, 2009). Biological activity of several 3-hydroxyquinoline-2,4-diones has been investigated (Prisyazhnyuk *et al.*, 1984; Luo *et al.*, 2009). 3-Alkyl- and 3-aryl-3-hydroxyquinoline-2,4-diones are important synthetic intermediates for the preparation of new types of heterocyclic compounds.

The asymmetric unit of the title compound (I) consists of a single 3-ethyl-3-hydroxy-8-methoxyquinoline-2,4(1*H*,3*H*)-dione molecule and solvated water molecule (Fig. 1). The piperidine ring that is fused to the benzene ring has a sofa shape with chiral carbon center 0.4084 (18) Å out of the plane for 9 ring atoms. In the crystal packing organic molecules and solvated water molecules are connected by two intermolecular O—H···O and one N—H···O hydrogen bonds. These connections altogether with additional O—H···O hydrogen bonding between hydroxyl and carbonyl groups (Table 1) form linear chain running along the *b* axis. Non–covalent π–π interactions occur between inversion–related benzene rings [centroid–centroid distance = 3.8846 (9) Å], which stabilize the crystal packing and connect the chains into a two-dimensional supramolecular layer parallel to the *bc* plane (Fig. 2).

### Experimental

To a solution of 3-ethyl-4-hydroxy-8-methoxyquinolin-2(1*H*)-one (4.38 g, 20.0 mmol) in 0.5 *M* sodium hydroxide (120 ml), 30% peroxyacetic acid in acetic acid (6.7 ml, 30 mmol) was added drop-wise within 30 min under stirring at room temperature. After several minutes a precipitate started to form. The reaction mixture was stirred for additional 30 min and then left at 10 °C overnight. The solid phase was filtered off under suction, dispersed in 5% aqueous sodium bicarbonate solution (25 ml), filtered off and washed with water (3 *x* 20 ml). Crystallization of the above air-dried solid from toluene afforded 3.92 g (83%) of the final product (I), *M*.pt 371–372 K.

### Refinement

The N-bonded hydrogen atom was located in a difference map and refined with the using distance restraint with N—H = 0.86±0.02 Å and with *U*_iso_(H) = 1.2*U*_eq_(N). Water H atoms were located in a difference map and refined with *U*_iso_(H) = 1.2*U*_eq_(O). The O—H distance of water were restrained to be 0.96±0.02 Å. All other H atoms were included in the model at geometrically calculated positions and refined using a riding model, with C—H bond lengths constrained to 0.93 Å (aromatic H), 0.96 Å (methyl H), 0.97 Å (methylene H) and O—H = 0.82 Å, and with *U*_iso_(H) values of 1.2*U*_eq_(C) [for aromatic and methylene H] or 1.5*U*_eq_(C) [for oxygen and methyl H].

### Crystal data

**Table:**

C_12_H_13_NO_4_·H_2_O	*D*_x_ = 1.388 Mg m^−^^3^
*M**_r_* = 253.25	Melting point = 371–372 K
Orthorhombic, *P**b**c**a*	Mo *K*α radiation, λ = 0.71073 Å
Hall symbol: -P 2ac 2ab	Cell parameters from 3161 reflections
*a* = 16.5055 (4) Å	θ = 1.0–27.5°
*b* = 8.8068 (2) Å	µ = 0.11 mm^−^^1^
*c* = 16.6690 (4) Å	*T* = 293 K
*V* = 2423.02 (10) Å^3^	Prism, yellow
*Z* = 8	0.50 × 0.25 × 0.20 mm
*F*(000) = 1072	

### Data collection

**Table:**

Nonius KappaCCD area-detector diffractometer	2779 independent reflections
Radiation source: fine-focus sealed tube	1963 reflections with *I* > 2σ(*I*)
Graphite monochromator	*R*_int_ = 0.019
φ scans + ω scans	θ_max_ = 27.5°, θ_min_ = 3.4°
Absorption correction: multi-scan (*SCALEPACK*; Otwinowski & Minor, 1997)	*h* = −21→21
*T*_min_ = 0.948, *T*_max_ = 0.979	*k* = −11→11
5200 measured reflections	*l* = −21→21

### Refinement

**Table:**

Refinement on *F*^2^	Primary atom site location: structure-invariant direct methods
Least-squares matrix: full	Secondary atom site location: difference Fourier map
*R*[*F*^2^ > 2σ(*F*^2^)] = 0.044	Hydrogen site location: inferred from neighbouring sites
*wR*(*F*^2^) = 0.127	H atoms treated by a mixture of independent and constrained refinement
*S* = 1.03	*w* = 1/[σ^2^(*F*_o_^2^) + (0.0662*P*)^2^ + 0.4669*P*] where *P* = (*F*_o_^2^ + 2*F*_c_^2^)/3
2779 reflections	(Δ/σ)_max_ = 0.0001
175 parameters	Δρ_max_ = 0.26 e Å^−^^3^
3 restraints	Δρ_min_ = −0.23 e Å^−^^3^

### Special details

**Table:**

Experimental. 211 frames in 4 sets of φ scans + ω scans. Rotation/frame = 2 °. Crystal-detector distance = 31 mm. Measuring time = 20 s/°.
Geometry. All s.u.'s (except the s.u. in the dihedral angle between two l.s. planes) are estimated using the full covariance matrix. The cell s.u.'s are taken into account individually in the estimation of s.u.'s in distances, angles and torsion angles; correlations between s.u.'s in cell parameters are only used when they are defined by crystal symmetry. An approximate (isotropic) treatment of cell s.u.'s is used for estimating s.u.'s involving l.s. planes.
Refinement. Refinement of *F*^2^ against ALL reflections. The weighted *R*-factor *wR* and goodness of fit *S* are based on *F*^2^, conventional *R*-factors *R* are based on *F*, with *F* set to zero for negative *F*^2^. The threshold expression of *F*^2^ > 2σ(*F*^2^) is used only for calculating *R*-factors(gt) *etc*. and is not relevant to the choice of reflections for refinement. *R*-factors based on *F*^2^ are statistically about twice as large as those based on *F*, and *R*- factors based on ALL data will be even larger.

### Fractional atomic coordinates and isotropic or equivalent isotropic displacement parameters (Å^2^)

**Table:**

	*x*	*y*	*z*	*U*_iso_*/*U*_eq_	
O1	0.08014 (8)	0.56475 (12)	0.75104 (6)	0.0505 (3)	
O2	0.04557 (8)	0.18242 (13)	0.56925 (8)	0.0576 (4)	
O3	0.05871 (8)	0.26982 (13)	0.72594 (7)	0.0527 (3)	
H3	0.0359	0.1965	0.7058	0.079*	
O4	0.14902 (8)	0.81485 (13)	0.51080 (7)	0.0542 (3)	
N1	0.11348 (8)	0.61105 (15)	0.62211 (7)	0.0395 (3)	
H1N	0.1065 (10)	0.7068 (16)	0.6299 (11)	0.047*	
C1	0.09586 (9)	0.51728 (17)	0.68405 (8)	0.0374 (3)	
C2	0.10269 (9)	0.34757 (17)	0.66669 (9)	0.0392 (4)	
C3	0.07607 (9)	0.30617 (16)	0.58203 (10)	0.0408 (4)	
C4	0.09477 (9)	0.41692 (17)	0.51912 (9)	0.0381 (3)	
C5	0.09051 (10)	0.3782 (2)	0.43780 (10)	0.0481 (4)	
H5	0.0777	0.2795	0.4227	0.058*	
C6	0.10534 (11)	0.4863 (2)	0.38047 (10)	0.0527 (4)	
H6	0.1023	0.4608	0.3264	0.063*	
C7	0.12485 (10)	0.6336 (2)	0.40285 (9)	0.0492 (4)	
H7	0.1344	0.7062	0.3634	0.059*	
C8	0.13022 (9)	0.67423 (18)	0.48253 (9)	0.0410 (4)	
C9	0.11398 (9)	0.56549 (17)	0.54186 (8)	0.0358 (3)	
C10	0.19372 (10)	0.3023 (2)	0.67026 (10)	0.0523 (4)	
H10A	0.1986	0.1950	0.6581	0.063*	
H10B	0.2228	0.3579	0.6291	0.063*	
C11	0.23315 (12)	0.3326 (3)	0.74990 (11)	0.0683 (6)	
H11A	0.2035	0.2817	0.7915	0.102*	
H11B	0.2333	0.4399	0.7602	0.102*	
H11C	0.2879	0.2957	0.7490	0.102*	
C12	0.16490 (14)	0.9320 (2)	0.45424 (12)	0.0670 (6)	
H12A	0.1179	0.9477	0.4214	0.101*	
H12B	0.2099	0.9033	0.4210	0.101*	
H12C	0.1777	1.0243	0.4822	0.101*	
O1W	0.06321 (11)	0.90745 (16)	0.66799 (10)	0.0752 (5)	
H1W	0.0478 (14)	0.978 (3)	0.6301 (13)	0.090*	
H2W	0.0132 (11)	0.900 (3)	0.6914 (14)	0.090*	

### Atomic displacement parameters (Å^2^)

**Table:**

	*U*^11^	*U*^22^	*U*^33^	*U*^12^	*U*^13^	*U*^23^
O1	0.0709 (8)	0.0477 (7)	0.0329 (6)	0.0008 (5)	0.0073 (5)	−0.0009 (5)
O2	0.0692 (8)	0.0379 (6)	0.0657 (8)	−0.0052 (5)	−0.0121 (6)	−0.0034 (5)
O3	0.0674 (8)	0.0436 (6)	0.0472 (7)	−0.0100 (6)	0.0055 (6)	0.0084 (5)
O4	0.0786 (9)	0.0454 (6)	0.0386 (6)	−0.0116 (6)	0.0060 (6)	0.0066 (5)
N1	0.0543 (8)	0.0341 (6)	0.0301 (6)	−0.0024 (5)	0.0009 (5)	−0.0008 (5)
C1	0.0403 (8)	0.0402 (8)	0.0318 (7)	0.0000 (6)	0.0002 (6)	0.0013 (6)
C2	0.0440 (8)	0.0380 (8)	0.0357 (8)	0.0022 (6)	0.0002 (6)	0.0046 (6)
C3	0.0398 (8)	0.0368 (8)	0.0457 (9)	0.0065 (6)	−0.0034 (7)	−0.0034 (6)
C4	0.0369 (7)	0.0427 (8)	0.0346 (7)	0.0054 (6)	−0.0030 (6)	−0.0040 (6)
C5	0.0512 (9)	0.0533 (10)	0.0399 (9)	0.0082 (8)	−0.0058 (7)	−0.0118 (7)
C6	0.0588 (10)	0.0682 (11)	0.0312 (8)	0.0108 (9)	−0.0013 (7)	−0.0079 (8)
C7	0.0531 (9)	0.0626 (11)	0.0319 (8)	0.0079 (8)	0.0049 (7)	0.0053 (7)
C8	0.0429 (8)	0.0451 (9)	0.0349 (8)	0.0033 (6)	0.0040 (6)	0.0024 (7)
C9	0.0360 (7)	0.0418 (8)	0.0295 (7)	0.0037 (6)	−0.0001 (6)	0.0000 (6)
C10	0.0496 (9)	0.0584 (10)	0.0490 (10)	0.0130 (8)	−0.0069 (8)	0.0009 (8)
C11	0.0583 (11)	0.0913 (16)	0.0553 (11)	0.0106 (11)	−0.0155 (9)	−0.0012 (10)
C12	0.0872 (15)	0.0552 (11)	0.0587 (12)	−0.0077 (10)	0.0127 (10)	0.0164 (9)
O1W	0.0992 (11)	0.0548 (8)	0.0715 (10)	0.0047 (8)	0.0365 (9)	0.0061 (7)

### Geometric parameters (Å, º)

**Table:**

O1—C1	1.2202 (17)	C6—C7	1.388 (3)
O2—C3	1.2193 (19)	C6—H6	0.9300
O3—C2	1.4040 (18)	C7—C8	1.378 (2)
O3—H3	0.8200	C7—H7	0.9300
O4—C8	1.361 (2)	C8—C9	1.402 (2)
O4—C12	1.422 (2)	C10—C11	1.502 (2)
N1—C1	1.3537 (19)	C10—H10A	0.9700
N1—C9	1.3966 (18)	C10—H10B	0.9700
N1—H1N	0.861 (14)	C11—H11A	0.9600
C1—C2	1.527 (2)	C11—H11B	0.9600
C2—C3	1.522 (2)	C11—H11C	0.9600
C2—C10	1.555 (2)	C12—H12A	0.9600
C3—C4	1.465 (2)	C12—H12B	0.9600
C4—C9	1.399 (2)	C12—H12C	0.9600
C4—C5	1.399 (2)	O1W—H1W	0.923 (16)
C5—C6	1.371 (3)	O1W—H2W	0.916 (16)
C5—H5	0.9300		
			
C2—O3—H3	109.5	C8—C7—H7	119.5
C8—O4—C12	118.23 (14)	C6—C7—H7	119.5
C1—N1—C9	123.82 (13)	O4—C8—C7	125.75 (15)
C1—N1—H1N	117.0 (12)	O4—C8—C9	114.89 (13)
C9—N1—H1N	115.2 (12)	C7—C8—C9	119.34 (15)
O1—C1—N1	122.32 (14)	N1—C9—C4	121.80 (13)
O1—C1—C2	121.64 (13)	N1—C9—C8	118.71 (13)
N1—C1—C2	115.90 (12)	C4—C9—C8	119.41 (13)
O3—C2—C3	112.71 (13)	C11—C10—C2	114.01 (15)
O3—C2—C1	107.81 (12)	C11—C10—H10A	108.8
C3—C2—C1	112.89 (12)	C2—C10—H10A	108.8
O3—C2—C10	110.35 (13)	C11—C10—H10B	108.8
C3—C2—C10	104.65 (12)	C2—C10—H10B	108.8
C1—C2—C10	108.35 (13)	H10A—C10—H10B	107.6
O2—C3—C4	123.84 (15)	C10—C11—H11A	109.5
O2—C3—C2	119.69 (14)	C10—C11—H11B	109.5
C4—C3—C2	116.32 (13)	H11A—C11—H11B	109.5
C9—C4—C5	120.10 (14)	C10—C11—H11C	109.5
C9—C4—C3	118.46 (13)	H11A—C11—H11C	109.5
C5—C4—C3	121.38 (14)	H11B—C11—H11C	109.5
C6—C5—C4	119.83 (16)	O4—C12—H12A	109.5
C6—C5—H5	120.1	O4—C12—H12B	109.5
C4—C5—H5	120.1	H12A—C12—H12B	109.5
C5—C6—C7	120.20 (15)	O4—C12—H12C	109.5
C5—C6—H6	119.9	H12A—C12—H12C	109.5
C7—C6—H6	119.9	H12B—C12—H12C	109.5
C8—C7—C6	121.09 (16)	H1W—O1W—H2W	95 (2)
			
C9—N1—C1—O1	−165.15 (15)	C4—C5—C6—C7	0.2 (2)
C9—N1—C1—C2	19.1 (2)	C5—C6—C7—C8	0.4 (3)
O1—C1—C2—O3	22.8 (2)	C12—O4—C8—C7	−0.1 (3)
N1—C1—C2—O3	−161.35 (13)	C12—O4—C8—C9	−178.93 (16)
O1—C1—C2—C3	147.98 (15)	C6—C7—C8—O4	179.85 (16)
N1—C1—C2—C3	−36.19 (18)	C6—C7—C8—C9	−1.4 (2)
O1—C1—C2—C10	−96.60 (17)	C1—N1—C9—C4	0.9 (2)
N1—C1—C2—C10	79.22 (16)	C1—N1—C9—C8	177.65 (14)
O3—C2—C3—O2	−26.4 (2)	C5—C4—C9—N1	175.61 (14)
C1—C2—C3—O2	−148.87 (14)	C3—C4—C9—N1	−1.8 (2)
C10—C2—C3—O2	93.51 (17)	C5—C4—C9—C8	−1.1 (2)
O3—C2—C3—C4	157.87 (13)	C3—C4—C9—C8	−178.52 (14)
C1—C2—C3—C4	35.42 (18)	O4—C8—C9—N1	3.8 (2)
C10—C2—C3—C4	−82.20 (16)	C7—C8—C9—N1	−175.08 (14)
O2—C3—C4—C9	167.10 (15)	O4—C8—C9—C4	−179.40 (14)
C2—C3—C4—C9	−17.4 (2)	C7—C8—C9—C4	1.7 (2)
O2—C3—C4—C5	−10.3 (2)	O3—C2—C10—C11	−58.1 (2)
C2—C3—C4—C5	165.20 (14)	C3—C2—C10—C11	−179.62 (16)
C9—C4—C5—C6	0.1 (2)	C1—C2—C10—C11	59.70 (19)
C3—C4—C5—C6	177.47 (15)		

### Hydrogen-bond geometry (Å, º)

**Table:**

*D*—H···*A*	*D*—H	H···*A*	*D*···*A*	*D*—H···*A*
O3—H3···O1^i^	0.82	2.35	2.9430 (17)	130
N1—H1*N*···O1*W*	0.86 (1)	2.01 (2)	2.8438 (19)	163 (2)
O1*W*—H1*W*···O2^ii^	0.92 (2)	2.07 (2)	2.9425 (19)	158 (2)
O1*W*—H2*W*···O3^iii^	0.92 (2)	2.15 (2)	2.9401 (19)	144 (2)

Symmetry codes: (i) −*x*, *y*−1/2, −*z*+3/2; (ii) *x*, *y*+1, *z*; (iii) −*x*, *y*+1/2, −*z*+3/2.
